# Current themes in neuroimaging studies of reading

**DOI:** 10.1016/j.bandl.2013.02.002

**Published:** 2013-05

**Authors:** Cathy J. Price

**Affiliations:** Wellcome Trust Centre for Neuroimaging, UCL, London WC1N 3BG, United Kingdom

**Keywords:** Reading, Neuroimaging, fMRI, MEG, TMS, DTI

## Abstract

This editorial provides a summary of the highlights from 11 new papers that have been published in a special issue of Brain and Language on the neurobiology of reading. The topics investigate reading mechanisms in both adults and children. Several of the findings illustrate how responses in the left ventral occipito-temporal cortex, and other reading areas, change with learning, expertise and the task: In the early stages of reading acquisition, learning/expertise increases activation in reading areas as well as in an attentionally-controlled, learning circuit. In later stages, expertise and efficiency decrease activation within the reading network and increase anatomical connectivity. Special interest is given to a white matter tract (the vertical occipital fasciculus) that projects dorsally from the left occipito-temporal cortex to the posterior parietal lobe. This observation fits with a magnetoencephalography study showing how activity in the angular gyrus is influenced by early occipito-temporal activity; with angular gyrus activity contributing to inferior frontal activity. Overall, the papers within the special issue illustrate the wide range of different techniques that can be used to reveal the functional anatomy of reading and the time course of activity within the different reading pathways.

## 1 Introduction

This special issue of *Brain and Language* includes a collection of 11 papers that illustrate current issues in the neurobiology of reading, and how these issues can be investigated with different neuroimaging techniques such as functional Magnetic Resonance Imaging (*fMRI*), Magnetoencephalography (*MEG*), Transcranial Magnetic Stimulation (*TMS*) and Diffusion Tensor Imaging (*DTI*). Although the content of each paper was left entirely to those who agreed to contribute, four notable themes emerged: (1) the function and connectivity of the left ventral occipito-temporal cortex; (2) the effect of experience, strategy and performance on reading activation and white matter tracks; (3) investigations of top down influences from the inferior frontal cortex; and (4) searches for brain regions and networks that might be dedicated to reading.

## 2 The left ventral occipito-temporal cortex

This is a large area at the junction of the occipital and temporal lobes; lying lateral to the fusiform gyrus and medial to the posterior inferior temporal gyrus. Olulade, Flowers, Napolielo, and Eden (2013) provide a neat demonstration of how word selectivity emerges along the posterior to anterior gradient. During a feature detection task, fMRI activation was greater posteriorly (*y* = −68 to −84) for unfamiliar false fonts compared to familiar letter strings consistent with increased demands on perceptual processing while anteriorly (*y* = −58 to −26), activation became more word selective and less involved in viewing unfamiliar false fonts.

The left ventral occipito-temporal cortex and nearby white matter are known to be essential for reading. Yeatman, Rauschecker and Wandell (2013) used a combination of fMRI and white matter tractography to define the anatomical organization in this region. The peak fMRI reading activation in ventral occipital cortex is adjacent and lateral to the foveal representation in visual field maps VO-1 and VO-2; and ventral and medial to human MT on the lateral occipital surface. At least three different white matter pathways pass near the peak fMRI reading activation and may carry reading signals: (i) the inferior longitudinal fasciculus (ILF) that links occipital cortex to the anterior and medial temporal lobes; (ii) the inferior fronto-occipital fasciculus (IFOF) that links occipital cortex to the ventrolateral prefrontal cortex; and (iii) the vertical occipital fasciculus (VOF) that projects dorsally to the lateral occipital parietal junction including the posterior angular gyrus and lateral superior occipital lobe. The cortical endpoints of the VOF are particularly dense in the ventral reading regions making this an important white matter target for future investigations.

The temporal duration and sequence of reading activity from the left fusiform gyrus were investigated by Simos, Rezaie, Fletcher and Papanicolaou (2013) using Magnetoencephalography (MEG) as school children read pseudowords aloud. The authors found that early activity in the left fusiform gyrus contributes to subsequent activity in the superior temporal and angular gyri (approximately 250 ms after onset) but there was no evidence for direct functional links from the left fusiform to either the supramarginal gyrus or the inferior frontal gyrus within the first 500 ms of processing. Activity in the inferior frontal gyrus was fed by activity from both the superior temporal and angular gyri (but not the supramarginal gyrus), while activity in the left supramarginal gyrus interacted bidirectionally with that in the superior temporal gyrus.

## 3 The effect of reading experience, strategy and performance

Now that the set of regions involved in reading is pretty well agreed upon, a popular new approach is to consider how the engagement of these regions varies within and across individuals according to experience and context. Papers in the special issue addressed this perspective in terms of developmental differences in reading activation for: (i) children versus adults (Olulade et al., 2013); (ii) learning to read a new artificial script (Mei et al., 2013); (iii) better versus worse reading skills in young children (Pugh et al., 2013) or adults (Olulade et al., 2013); (iv) familiarity with the orthography in adults (Twomey et al., 2013); (v) word frequency in adults (Heim, Wehnelt, Grande, Huber, & Amunts, 2013); (vi) pseudoword versus word reading in adults (Heim et al., 2013); and (vii) task instructions in adults (Cummine, Gould, Zhou, & Hry, 2013). In addition, the special issue reports a paper showing the effect of reading performance on white matter pathways (Lebel et al., 2013).

Overall, the effect of experience is consistent with an inverted U-shaped function (Price & Devlin, 2011). The rising part of this inverted-U is illustrated by the studies showing that learning to read increases activation in mid-regions of the left ventral occipito-temporal cortex. Specifically, responses in this region are higher after adults learn to read a new script irrespective of whether learning focused on lexical or sublexical strategies (Mei et al., 2013). Pugh et al. (2013) also show higher activation in a wide network of reading areas for young children (5–8 years) who have better relative to worse performance on tasks tapping auditory sensorimotor processing, phonological awareness, pseudoword decoding and word reading ability. Increases in activation with better reading performance illustrate the development of functional connections between orthographic inputs and language areas. They may also reflect an attentionally-controlled, learning circuit that is more involved in children with better reading readiness skills (Pugh et al., 2013). The falling part of the inverted-U shape function is illustrated by studies showing that activation is higher for newly learnt or less familiar words than it is for familiar words that are frequently experienced. For example, activation in mid-regions of the left ventral occipito-temporal cortex decreases with the familiarity of the script (Twomey et al., 2013) and in adults relative to children (Olulade et al., 2013), and activation in inferior frontal areas decreases with increases in word frequency (Heim et al., 2013) and for words relative to pseudowords (Heim et al., 2013).

Over and above the inverted-U shaped learning function, activation varies with the reading strategy adopted. This is illustrated by Heim et al. (2013) in a comparison of frequency effects in adults with and without dyslexia Olulade et al. (2013) for adults with advanced word recognition skills and by Cummine et al. (2013) in a comparison of reading activation to the same words under different reading instructions. Heim et al. (2013) found that, in developmental dyslexics, lexicality effects (pseudowords > words) and frequency effects (low > high frequency) were weaker than controls in BA 45 (associated with lexical selection) but stronger than controls in BA44 (that supports lexical access via grapheme-to-phoneme conversion). The lexicality and frequency effects in BA 45 were even stronger in those adult controls who had overcome childhood dyslexia. Olulade et al. (2013) found that adults who have exceptionally good word recognition skills activate an anterior area in the parahippocampal gyrus that is not activated in other subjects when the task is feature detection. Meanwhile, Cummine et al. (2013) illustrate the effect of different reading strategies within the same individuals (skilled readers) on activation in response to seeing: (i) real words with regular spellings; (ii) real words with irregular spellings and (iii) pseudowords. Instructions to name only the words (ignoring pseudowords), compared to instructions to name all stimuli, increased the overlap between activation in the left middle temporal cortex for words with regular and irregular spellings. This suggests that subjects can be encouraged to read regular words using a lexical strategy (more likely when ignoring pseudowords) or a sublexical strategy (more likely when switching between words and pseudowords).

It is additionally interesting to note that better reading performance increases the fractional anisotropy (FA) of white matter particularly in the frontal lobes (Lebel et al., 2013). Thus, efficient reading in adults is typically associated with lower reading activation (Heim et al., 2013; Twomey et al., 2013) but increased anatomical connectivity within the reading network (Lebel et al., 2013). Plausibly, efficient reading, and lower reading activation for familiar words, might both be the consequence of increased connectivity within the reading network. However, it is also plausible that reading experience increases myelination, axon density, and/or axonal integrity which in turn facilitates future reading ability.

## 4 Top down influences from the inferior frontal cortex?

The demonstration that activation in the reading network increases during learning and decreases with familiarity can be explained by both bottom up and top down processing within the network. The contribution of top down processes is particularly evident when effects of word familiarity and reading strategy are observed in the context of controlling for the visual input or word frequency (Cummine et al., 2013; Twomey et al., 2013).

Two studies in the special issue directly investigate the top down influence of early inferior frontal lobe activity (Simos et al., 2013; Wheat et al., 2013). Simos et al., (2013) found no evidence for feedback influences from the inferior frontal, superior temporal or parietal regions to the fusiform gyrus. However, as acknowledged by the authors, the analysis of their MEG data may not be sufficient to detect activity modulations that might be small relative to bottom up effects, and therefore future studies using small time bins (e.g. 50 ms rather than 100 ms) are needed to further investigate top down effects. Wheat et al. (2013) were also unable to find evidence that early dorsal inferior frontal activation (within the first 200 ms after stimulation) contributed to reading performance because online Transcranial Magnetic Stimulation (TMS) did not disrupt reading in the first 225 ms after stimulus onset although TMS to the same site did slow both reading and picture naming responses between 225 and 300 ms. Wheat et al. (2013) therefore suggest that early inferior frontal activity, detected by MEG studies of phonological processing, could reflect activation that occurs implicitly during reading aloud even when it is not required by the task. Wheat et al. also acknowledge the possibility that early dorsal inferior frontal activation (within the first 200 ms of word processing) might contribute to performance during other tasks (e.g. lexical decision), contexts (reading novel or low frequency words) or languages (e.g. English words with opaque spellings versus Dutch words with transparent spellings). It is also possible that early activity in other frontal regions (e.g. left pars orbitalis) is necessary for reading aloud single high frequency words or naming pictures. For example, top down effects on posterior visual word processing areas may be coming from ventral rather than dorsal inferior frontal regions (Woodhead et al. (2013)) and the “necessity” of this activation for reading could be tested by future TMS studies.

## 5 What is unique about the reading network?

Two papers in the special issue remark on the absence of any evidence for neural responses that are unique to reading (Vogel et al., 2013; Wheat et al., 2013). Wheat et al. (2013) note that the results of their TMS study demonstrate that the timing and location of dorsal inferior frontal activation is indistinguishable for reading and picture naming. Vogel and colleagues take this a step further by reporting no evidence for networks of regions that are dedicated to reading. Their conclusions are based on a resting state functional connectivity analysis of fMRI data with the rationale that correlated activity at rest reflects the history of co-activation. Thus, if reading regions typically activate with other reading regions but rarely with non-reading regions, then activity in reading regions would be tightly correlated at rest. Alternatively, if reading regions were occasionally activated together (during reading), but more frequently activated with other regions (during non-reading tasks), then activity in reading regions would not be tightly correlated at rest. The results showed the latter with reading related regions (e.g. ventral occipito-temporal cortex, middle temporal gyrus, angular gyrus, etc.) distributed among a number of different networks. The authors therefore conclude that there was no indication of a specific community of brain regions that is devoted to reading, and no indication that such a community emerges with development. Reading-related regions are therefore likely to perform functions that are useful for, but not specific to, reading.

## 6 Summary

This special issue on the neurobiology of reading provides a heterogeneous collection of papers that use sophisticated experimental designs, acquisitions and analyses to provide a deeper understanding of reading mechanisms. In addition to the themes described above, each paper provides useful literature reviews, methodological ideas and a host of new results that will contribute to shaping future investigations into the neural systems that support reading.
